# Dynamics of zoonotic soil-transmitted helminth infections in wild African green monkey (*Chlorocebus aethiops sabaeus*)

**DOI:** 10.1017/S0031182026102212

**Published:** 2026-06

**Authors:** Yun Teng, Lee Zhen Lin, Christina Perea, Chaoqun Yao

**Affiliations:** Department of Biomedical Sciences, Ross University School of Veterinary Medicine, Basseterre, Saint Kitts and Nevis

**Keywords:** African green monkey, *Ancylostoma duodenale*, *Chlorocebus aethiops sabaeus*, hookworm, *Necator americanus*, soil-transmitted helminths

## Abstract

One quarter of humans are infected with soil-transmitted helminths (STHs) worldwide. African green monkey (AGM, *Chlorocebus aethiops sabaeus*) interacts directly or indirectly with humans frequently on the island of St Kitts. They serve as a reservoir for zoonotic pathogens of significant public health concerns. We have previously demonstrated that AGMs are infected with zoonotic parasites *Trichuris trichiura* and *Hymenolepis diminuta*. Nevertheless, research on their STHs is limited. In this study, faeces were opportunistically collected from wild AGMs near residential areas between May 2020 and March 2022. They were examined for zoonotic STHs using direct smears (2 per sample) and molecular detection (PCR and DNA sequencing). The positive detection rates for whipworm were 60.0%, 60.0% for individual smears and 77.8% when both smears were combined. For hookworm, the rates were 13.3%, 6.7%, and 15.6%, respectively. Agreement between direct smear and molecular detection was 44.4% for whipworm and 88.9% for hookworm. The study further compared the sensitivity of direct smear, flotation and molecular detection, demonstrating that prolonged deep freezing (−80 °C) minimally alters egg morphology but markedly impairs egg flotation, whereas PCR and direct smear remain informative for frozen faecal samples. DNA sequencing of the internal transcribed spacers identified the hookworm as *Ancylostoma duodenale* and *Necator americanus*, the human hookworms, for the first time genetically in AGMs. These findings underscore the need to include AGMs as an integral component of One Health strategies aiming at eliminating STH morbidity in pre-school and school-age children on St Kitts and other Caribbean islands.

## Graphical abstract

**Figure:**
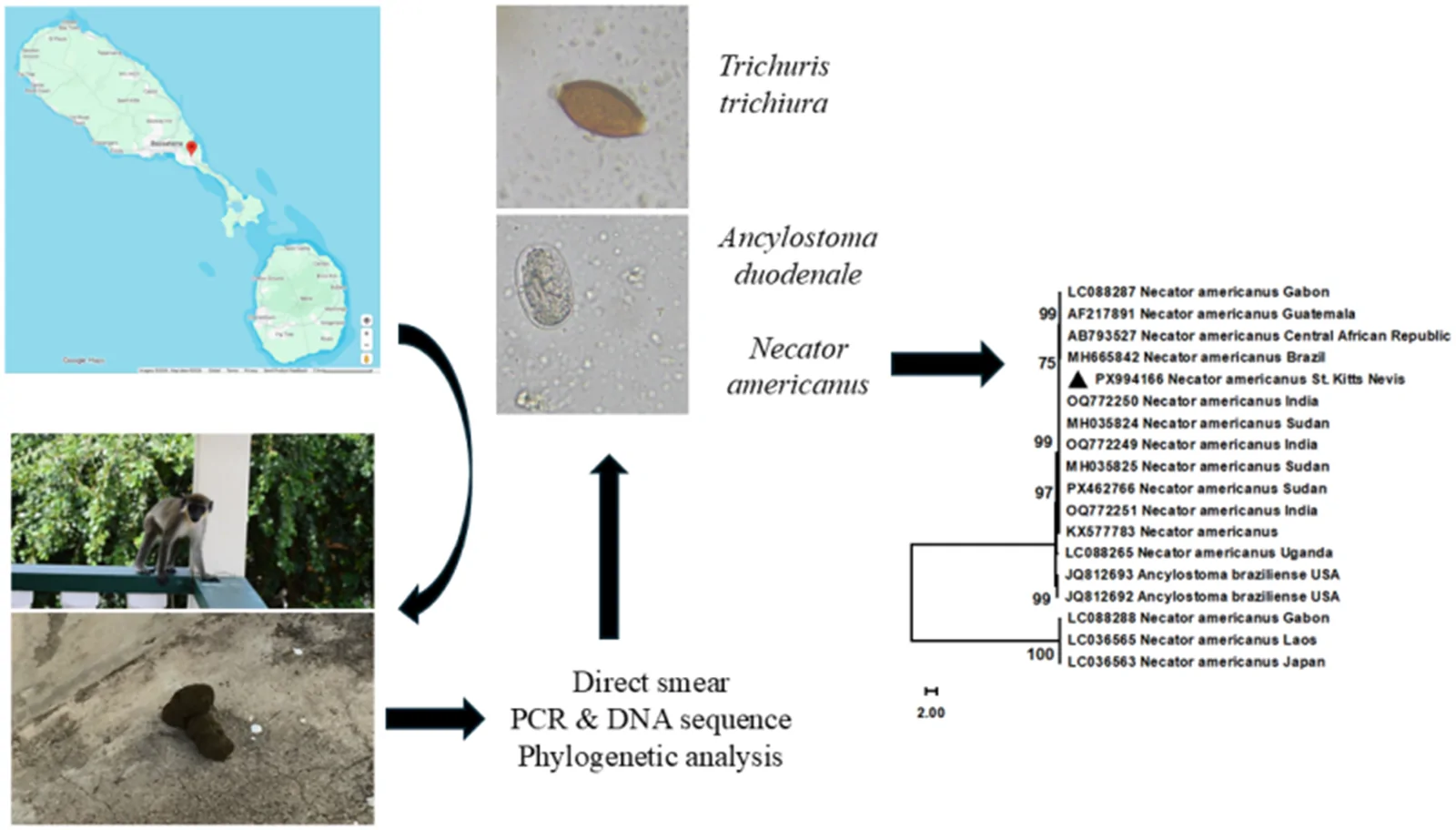

## Introduction

African green monkey (AGM, *Chlorocebus aethiops sabaeus*) is an invasive species to the island of St Kitts that was introduced from Africa in the 17th century. It has adapted well to this exotic home in the Caribbean since its introduction. By estimate, its present population is approximately equal to the number of human residents on the island, and it plays a role in the local economy both as an attraction for tourists and as a pet (Yao et al., 2018b; Yao, 2023). AGMs on St Kitts have been previously found to be infected with several zoonotic parasites. They are *Trichuris trichiura* (Yao et al., 2018b), *Hymenolepis diminuta* (Yao, 2023), *Strongyloides fuelleborni* (Richins et al., 2023) and *Schistosoma mansoni* (Ketzis et al., 2020; Eckhaus et al., 2025). Furthermore, the prevalence of *T. trichiura* and *H. diminuta* was reported to be 91% and 20%, respectively (Yao et al., 2018b; Yao, 2023).

According to the World Health Organization (WHO), soil-transmitted helminths (STHs) infect approximately 1.5 billion people, or 24% of the world’s population. One of the 6 global targets of the WHO is to eliminate STH morbidity in pre-school and school-age children (https://www.who.int/soil-transmitted-helminth-infections, accessed on 08 December 2025). For Kittitian school children, the latest available data on STH infections were from a study carried out in 1982 and 1991. Five- and 9-year-old children were the research subjects. More than 50% of children were found infected with at least 1 STH in both years (Rawlins et al., 1993). Unfortunately, the latest data are unavailable for populations, including school-age children on St Kitts, since the 1990s.

AGMs likely play a pivotal role in STHs transmission and prevalence on St Kitts. However, no studies have examined the longitudinal or seasonal dynamics of STHs in wild AGM populations. This is mainly due to logistical challenges to collect faecal samples from wild AGMs for an extended period. We hypothesized that AGMs’ infections by STHs on St Kitts showed no seasonality based on the tropical weathers with monthly average temperatures in winter over 75 ^o^F. Hence, they posed risks to humans year-round. Furthermore, we would like to fill the gap of identifying the species of hookworms infecting AGMs since this has never been done. AGM’s faeces were tested for whipworms (*T. trichiura*) and hookworms (*Ancylostoma duodenale* and *Necator americanus*) by direct smear, flotation and molecular techniques (PCR and DNA sequencing). The data generated in the current study lay a solid foundation for developing a comprehensive strategy to control and manage STHs on St Kitts using a One-Health approach.

## Materials and methods

### Study location and seasonality

**Figure 1.**
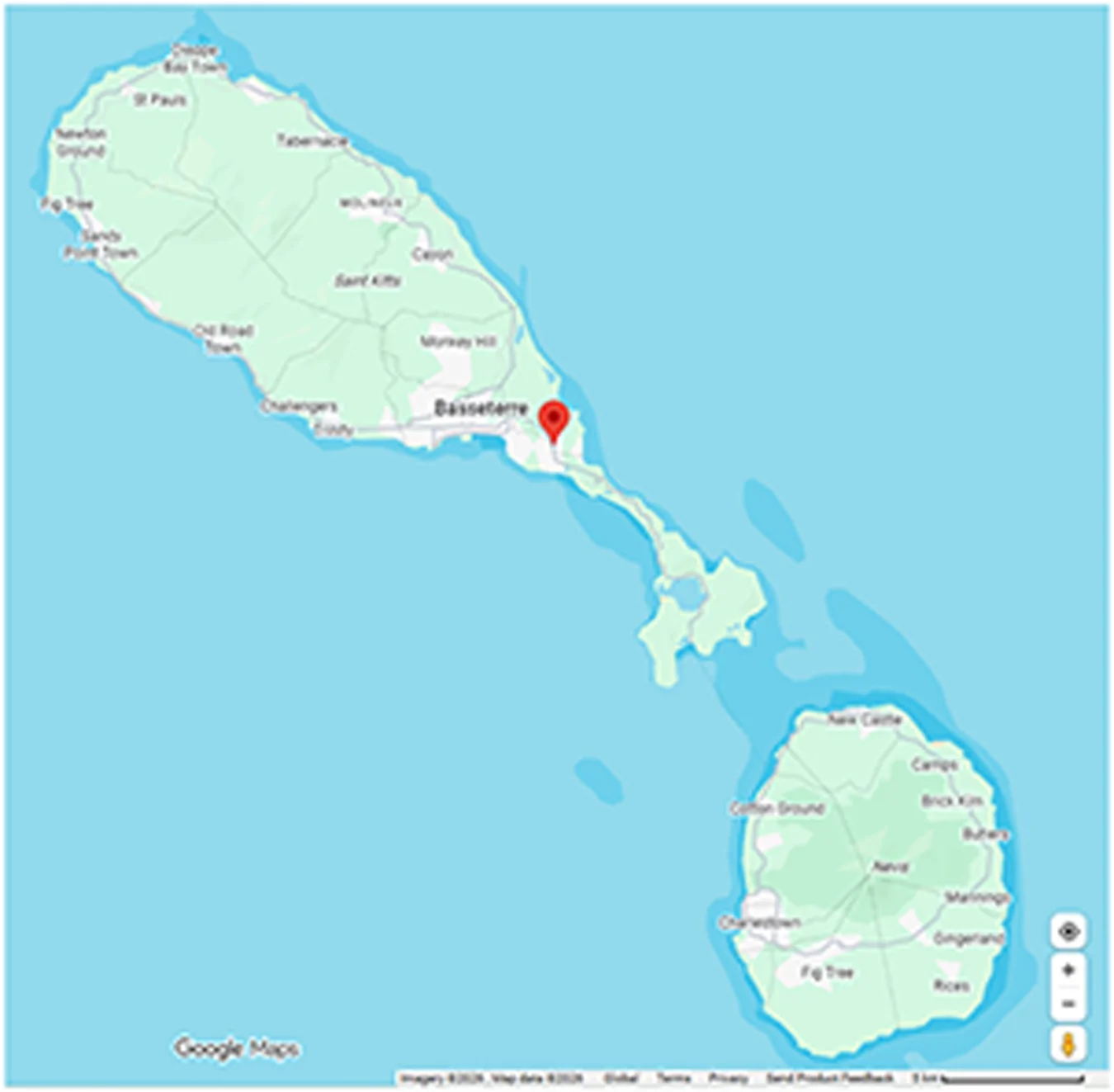
Sample collection sites in St Kitts and Nevis. The red mark shows the location by Google Map where all faeces of African green monkeys used in the current study were collected. Figure 1 long description.

The study was carried out in St Kitts and Nevis, an inland country in the Caribbean (17^o^20′N 62^o^45′W, https://www.gov.kn/about-st-kitts-and-nevis/, accessed on 13 April 2026) with a national population of 50 726. The federation consists of 2 islands, St Kitts (69 sq. miles) and Nevis (36 sq. miles). The location of faecal sample collection was Frigate Bay at St Kitts (Figure 1, by Google Map). St Kitts has a tropical weather, with monthly average temperatures in spring (March to May) 78–81 ^o^F, summer (June to August) 82–83 ^o^F, fall (September to November) 81–83 ^o^F and winter (December to February) 78–79 ^o^F (Supplementary Table 1). Alternatively, the Kittitian weather shows dry and wet seasons of tropical weather, roughly ranging from December to April and May to November, respectively (Average-Weather-in-Saint-Kitts-and-Nevis, accessed on 02 September 2025).

### Faeces collection

**Figure 2.**
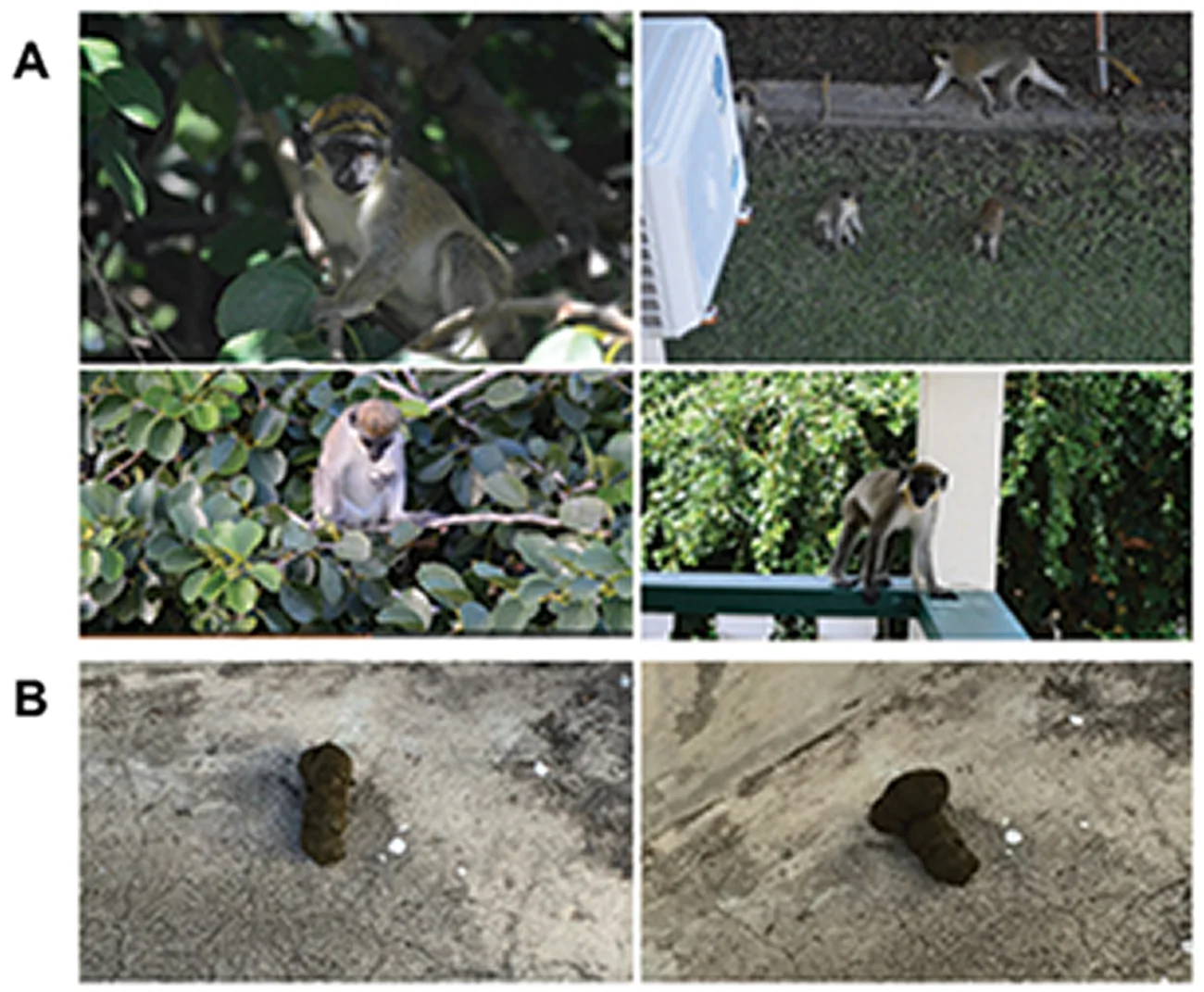
African green monkeys (AGMs) and their faeces. (A) Members of the troop of wild AGMs, whose faeces were collected and used in the study. (B) AGMs faeces laid on concrete surfaces. These were not from the AGMs that their faeces were used in the study. The pictures are only used for illustration purposes to show faeces on the concrete surface, similar to those used in the current study. Figure 2 long description.

All AGM’s faecal samples used in the current study were opportunistically collected by the senior author (C.Y.) from cement grounds between March 2020 and March 2022 in a fenced yard of a house located in Frigate Bay, Basseterre, St Kitts and Nevis. A troop of wild AGMs frequently entered the yard and occasionally accessed the second-floor balcony (Figure 2). Fresh faecal deposits on cement surfaces were collected within minutes of defecation using a clean, inside-out Ziplock plastic sandwich bag (Johnson & Son Inc., Racine, WI) worn on the right hand. Each sample was immediately flattened to approximately 3–5 mm thickness to facilitate rapid freezing and placed in a household freezer at −18 to −20 °C (Frigidaire Appliance Company, Charlotte, NC). Samples were subsequently transported on ice to the campus of Ross University School of Veterinary Medicine, typically within 1–2 months of collection, and stored at −80 °C until analysis. Because all samples were collected non-invasively from wild AGMs without direct animal handling or experimental manipulation, Institutional Animal Care and Use Committee (IACUC) approval was not required.

### Direct smear

For the detection of intestinal parasites, individual faeces were subjected to direct smear for morphologic identification of STHs, including whipworm and hookworm. Although direct smear is of limited sensitivity, it was chosen over centrifugal flotation because all faecal samples used in the study were kept in −80 °C for many months prior to test. As discussed later, many parasites, including eggs of whipworms and hookworms, the main target parasites of the study, do not float even in Sheather’s sugar solution with centrifugation. Approximately 0.1 g of frozen faeces was homogenized with a wooden toothpick in 2 drops of phosphate-buffered saline (pH 7.2) upon thawing at room temperature on a glass slide. A coverslip was added to cover the faecal smear. Clear nail polish was applied to seal 4 edges of the cover slip to prevent slide from quickly drying up during microscopic examination. Duplicate smears were used for each faecal sample. The slides were examined under a Nikon microscope (Nikon Instruments, Inc., Melville, NY) equipped with an Olympus DP27 digital camera (Olympus Corporation of the Americas, Breinigsville, PA). Eggs were identified by morphology and photographed at both 10× and 40× objective lenses. Sensitivity of individual faecal smears was calculated as: (positive number of each faecal smear/total positive number of both smears combined) × 100%.

### Flotation by double centrifugation

Double centrifugal faecal flotation was performed following the standard protocol outlined by Zajac et al. (2021). Briefly, approximately 2 g of individual faeces were homogenized in distilled water in 15 mL conical centrifuge tubes (Fisher Scientific, Pittsburgh, PA) and centrifuged at 500 *× g* for 5 min at room temperature in the first centrifugation. The Sheather’s sugar solution was used in the second centrifugation with a cover slip placed at the top of tubes under the same centrifugation. The slides were microscopically examined immediately as outlined in the previous paragraph. Eggs per gram of faeces (EPG) of individual faeces were recorded.

### Faecal DNA extraction

Total DNA was extracted from individual faeces using QIAamp DNA Stool Kit (QIAGEN, Hilden, Germany) following the supplier’s protocol with a minor modification, which was to increase the initial incubation temperature from 70 to 95 °C to break the hardy eggs such as *T. trichiura* as previously described (Yao et al., 2018b). Quality and quantity of the extracted DNA were monitored using a NanoPhotometer (Implen, Inc., Westlake Village, CA). DNAs were stored at − 20 °C until use.

### PCR positive controls

One positive control was used for each of 3 STHs in PCR. For *T. trichiura*, the purified PCR amplicon of 18S rRNA (accession number KX961639, 498 bp) was used (Yao et al., 2018b). Internal transcribed spacers (ITS) of *A. duodenale* (MK271367, 704 bp) and *N. americanus* (LC036563, 1290 bp) were synthesized by Integrated DNA Technologies, Inc. (IDT, Coralville, Iowa). In titration using primer to DNA ratios of 1:10^−5^–10^−16^ µM, conventional PCR consistently detected target DNA in 3 independent repeats across 6 orders of magnitude from 10^−5^ µM to 10^−10^ µM (Supplementary Figure 1 and Table 2 in Supplementary – Primer to DNA ratio). Consequently, positive controls were set at 10^−10^ µM, the lowest DNA concentration.

Each faecal DNA was first subjected to PCR amplification of AGM-*nadh1* in conventional PCR to rule out PCR inhibitors in DNA preparations as previously described (Yao et al., 2018b), which served as an internal control. Additional DNA extracts were prepared from the faeces that their first DNAs yielded no AGM-*nadh1* after 2 attempts. The same PCR was performed for the 2nd DNA extracts. If the 2nd DNA failed to yield amplicons for AGM-*nadh1* upon 2 attempts as well, these DNAs would be excluded from PCR to detect whipworm and hookworm since they very likely contained PCR inhibitors.

### PCR and DNA sequencing

The PCR targets, primer sequences and expected size of amplicons of whipworm and hookworm are listed in Table 1. All primers were synthesized by Integrated DNA Technologies, Inc. (IDT, Coralville, IA).

**Table 1.** Characteristics of conventional PCRs targeting whipworm and hookworm Table 1 long description.

Species	Target gene	Primer sequence	Expected size (bp)	PCR mix^a^	Thermal cycle	Reference
African green monkey	*nadh1*	CGCCCTAATGGAGCCCTACGAGCCG TAGAAAGATTGTAGTTGTTAGGGCG	348	T1	95^o^C 2 m, 35 cycles (95 °C 30s, 55 °C 1 m, 72 °C 1 m), 72 °C 10 m	(Yao et al., 2018b)
*Trichuris trichiura*	18S rRNA	CTGCGAGGATTGACAGATCA GTACAAAGGGCAGGGACGTA	498	T2	As above	(Yao et al., 2018b)
*Necator americanus*	ITS1−2	GATGAGCATTGCWTGAATGCCG GCAAGTRCCGTTCGACAAACAG	483	T2	As above except annealing at 64 °C	(Traub et al., 2008)
*Ancylostoma duodenale*	ITS1−2	GATGAGCATTGCWTGAATGCCG GCAAGTRCCGTTCGACAAACAG	380	T2	As above except annealing at 64 °C	(Traub et al., 2008)

a T1: HotStart Taq Plus 2 × Master Mix (QIAGEN); T2: Taq DNA polymerase (TaKaRa), 0.2 mM dNTPs, 2.0 mM MgCl_2_.

For PCRs, 2 types of PCR mix were used with a fixed concentration of 1.0 µM for each primer. These were HotStart Taq Plus 2× Master Mix (QIAGEN) and Taq DNA polymerase (TaKaRa, Clontech, CA; Table 1). In the latter, 0.2 mM dNTPs and 2.0 mM MgCl_2_ were used. All PCR reactions were carried out in a Mastercycler Nexus Gradient thermal cycler (Eppendorf, Enfield, CT). Each PCR included both positive and negative controls. Positive control DNAs were set at 10^−10^ µM as described in the previous section, and negative controls were molecular-grade water. PCR amplicons were detected by gel electrophoresis in 1.2% agarose. A total of 100-bp DNA ladder (Invitrogen, Carlsbad, CA) was used.

PCR amplicons of expected size for hookworms were purified from gel slices using QIAquick Gel Extraction Kit (QIAGEN). The purified PCR amplicons were directly sequenced in both directions using PCR primers (Macrogen, Seoul, Korea). Consensus DNA sequences were built from both sequences with occasional manual read-out of sequencing chromatogram for confirmation if needed. The resultant DNA sequences were used to search GenBank using BLAST (Basic Local Alignment Search Tool) on the NCBI website (https://blast.ncbi.nlm.nih.gov/Blast) for hookworm species identification.

### Phylogenetic analysis

ITS sequences of *A. duodenale* and *N. americanus* including the obtained ITS sequences (GenBank accession numbers: PX651763 and PX994166, respectively), *A. caninum* and *A. braziliense* in the GenBank were analysed using Molecular Evolutionary Genetics Analysis (MEGA, version 12.1) (Kumar et al., 2024). The most suitable substitution model for Maximum Likelihood method was first determined, which was Tamura 3-parameter (Tamura et al., 2011). The default settings were applied with 1000 bootstrap replications.

### Kappa agreement between direct smear and PCR

The kappa statistic was performed to determine the degree of agreement between double direct smears and PCR detection of whipworm and hookworm as previously described (McHugh, 2012). The level of agreement was determined by kappa values as follows: <0.39, minimal; 0.40–0.59, weak; 0.60–0.79, moderate; and >0.80, strong.

## Results

### Faecal sample collection

Forty-five faecal samples were opportunistically collected between March 2020 and March 2022 at a single location (Figure 1). Specifically, 8, 3, 26 and 8 samples were collected in spring (March to May), summer (June to August), fall (September to November) and winter (December to February), respectively.

### No seasonality of soil-transmitted-helminth infections in AGMs

#### Two direct smears increase test sensitivity greatly

**Figure 3.**
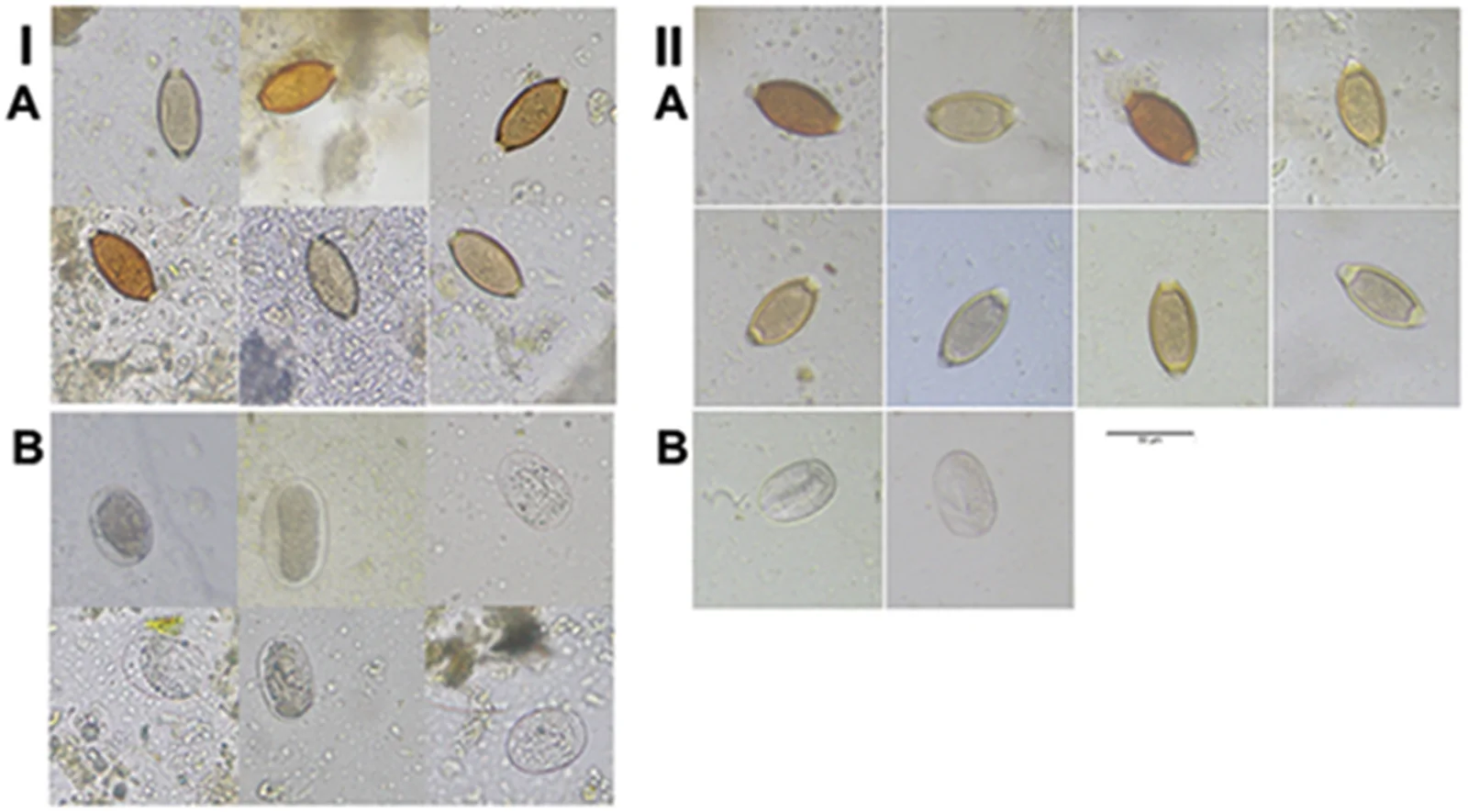
Eggs of whipworms and hookworms in African green monkeys (AGM). (I) Parasites detected in deep-frozen AGM’s faeces (−80 °C) by direct smear. (II) Parasites detected in fresh AGM’s faeces recovered by double centrifugal flotation. (A) Whipworm eggs. (B) Hookworm eggs. All images were taken under 40× objective lens. Scale bar: 50 µM. Figure 3 long description.

For each faecal sample, 2 direct smears were microscopically examined under 10× and 40× objective lenses. Two parasites were positively identified: whipworm and hookworm (Figure 3I A and B). The sensitivity of individual one smear for detecting whipworms was 77.1% and 77.1%. Similarly, the sensitivity for hookworms was 42.9% for one and 85.7% for the other (Table 2). Two smears together greatly boosted sensitivity of detection by 22.9% for whipworm and up to 57.1% for hookworm. The positive detection rates for whipworm were 60.0% (27/45) for the first smear, 60.0% (27/45) for the second smear and 77.8% (35/45) for the combination of both smears. For hookworm, the rates were 13.3% (6/45), 6.7% (3/45) and 15.6% (7/45), respectively (Table 2). Furthermore, all hookworm-positive faeces were also positive for whipworm. Collectively, the combined prevalence of both whipworm and hookworm by direct smear among all 45 faeces examined was 77.8% (35/45).

**Table 2.** Sensitivity and prevalence of whipworm and hookworm by direct smear among African green monkeys on St Kitts Table 2 long description.

Parasites	Sample no.	Smear #1 + (sensitivity)^a^	Smear #2 + (sensitivity)^a^	Both slides +	Total +	Prevalence^b^
Whipworm	45	8 (77.1%)	8 (77.1%)	19	35	77.8%
Hookworm	45	4 (85.7%)	1 (42.9%)	2	7	15.6%
Both	45	4 (85.7%)	1 (42.9%)	2	7	15.6%

a Sensitivity = (individual positive + both positive)/total positive no. × 100%.

b Prevalence = total positive numbers/sample no. × 100%.

#### PCR detection

Faecal DNAs were first tested for AGM *nadh1* by PCR to monitor whether PCR inhibitors existed. Only 27 of 42 samples remained after 2 rounds of PCR on each of 2 independently extracted faecal DNAs of individual samples. Altogether, PCR failure rate in amplification of AGM *nadh1* was 35.7% (15/42), indicating PCR inhibitors in those DNA preparations. At the end, these 27 DNAs (64.3%) were further used in PCR testing for whipworm and hookworm.

PCR targeting 18S rRNA of *T. trichiura* was positive for 2 of the 7 direct-smear negative faecal samples and negative for 13 of the 20 direct-smear positive faecal samples. Overall, PCR prevalence was 33.3% (9/27). The 2 test methods minimally agreed with one another (Kappa *K* = 0.04). PCR targeting ITS of hookworms would yield different sizes of amplicons, 483 bp for *N. americanus* and 380 bp for *A. duodenale* (Supplementary Figure 1 in Supplementary – Primer to DNA ratio). This PCR was positive for 2 of the 24 direct-smear-negative faecal samples and negative for 1 of the 3 direct-smear-positive faecal samples. Overall, the PCR prevalence of hookworm was 14.8% (4/27). The 2 test methods had Kappa *K* = 0.51, indicating a weak agreement between direct smear and PCR.

#### Seasonal infection variation

Among 45 faeces, 37 and 9 were detected positive for whipworm and hookworm by a combination of direct smear and PCR, respectively. The prevalence was 82.2% (95% CI 71.0–93.4%) and 20.0% (95% CI 8.3–31.7%), respectively.

Among the 45 faecal samples, 8, 3, 26 and 8 were collected in spring, summer, fall and winter, respectively. The infection rates of whipworm among the 4 seasons were 87.5% (7/8), 100.0% (3/3), 80.8% (21/26) and 75% (6/8), respectively (χ^2^, df = 3, *P*=0.77). The infection rates of hookworms were 12.5% (1/8), 33.3% (1/3), 19.2% (5/26) and 20.0% (2/8), respectively (χ^2^, df = 3, *P*=0.86). These data statistically showed minimal seasonal fluctuations for both parasitic infections in AGMs. The infection rates of whipworm in dry and wet seasons were 84.6% (11/13) and 81.3% (26/32), respectively (χ^2^, df = 1, *P*=0.79). The infection rates of hookworm were 23.1% (3/13) and 18.8% (6/32), respectively (χ^2^, df = 1, *P*=0.74). These data show that both whipworm and hookworm have similar prevalence year-round with little fluctuation.

### Parasite eggs subjected to deep frozen do not float even by centrifugal flotation

Two fresh (used in 2 days after collection) and 2 frozen faecal samples were used in double centrifugal flotation. They were from different collections rather than the same samples. One fresh faeces named F1 was collected at the campus of Ross University School of Veterinary Medicine and the other F2 at the same residential house as described earlier. Whipworm eggs were detected in 1 of 2 direct smears of both F1 and F2 faeces. Double centrifugal flotation yielded whipworm eggs from both F1 and F2, and hookworm eggs from only F2 (Figure 3II A and B). EPG was 8.3 for F1 and 17.9 for F2 for whipworm. The frozen sample #13 (frozen for 33 months after collection) was found to have whipworm eggs on both direct smears, and #19 (frozen for 42 months after collection) was found to have whipworm eggs on 1 of 2 direct smears and PCR positive for hookworms. However, not a single egg of whipworm nor hookworm was detected by flotation for both frozen faeces, indicating deep-frozen whipworm and hookworm eggs do not float even in the Sheather’s sugar solution by centrifugal flotation.

### Hookworms infecting AGMs are Ancylostoma duodenale and Necator americanus

We have previously determined that whipworms infecting AGMs are *T. trichiura* by PCR and DNA sequencing (Yao et al., 2018b). However, hookworms infecting AGMs have not been identified to species. The same primers targeting hookworm ITS (Table 1) span *N. americanus* (485 pb) and *Ancylostoma* spp. (380 bp) (Supplementary Figure 1 in Supplementary – Primer to DNA ratio). PCR of individual faecal DNAs detected both, indicating that hookworms infecting AGMs were *Ancylostoma* sp. and *Necator* sp., respectively. Furthermore, one each of 380- and 480-pb PCR amplicons was subjected to DNA sequencing, which confirmed them as *A. duodenale* (GenBank accession number: PX651763) and *N. americanus* (GenBank accession number: PX994166) that originated from 2 different faecal samples upon BLAST.

**Figure 4.**
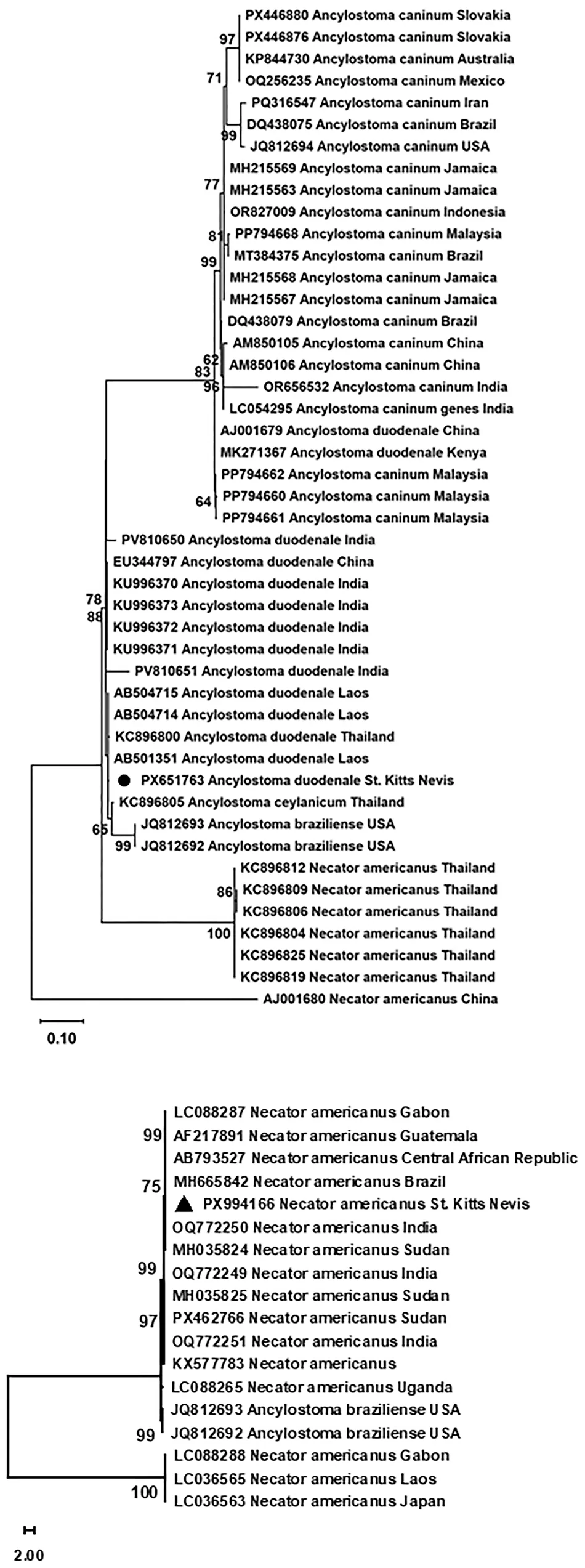
Phylogenetic analysis of the internal transcribed spacers of hookworms through Maximum Likelihood. The new DNA sequence is marked by a solid dot for *Ancylostoma* spp. (A) and a solid triangle for *Necator americanus.* (B) Phylogenetic analyses were performed using Molecular Evolutionary Genetics Analysis (MEGA, version 12). Scale shows the number of substitutions per site. Figure 4 long description.

Phylogenetic analysis showed that the new ITS sequence of *A. duodenale* reported here (PX651763) formed a clade with the homologous sequences of hookworms originated from Asian countries including China, India, Laos and Thailand. The clade also covered *A. ceylanicum* and *A. braziliense*. A sister clade was formed by *A. caninum* in Africa (Kenya), America (Brazil, Jamaica, Mexico and USA), Asia (China, India, Indonesia, Iran and Malaysia) and Australia. Interestingly, 2 entries of *A. duodenale* ITS, one each from China (AJ001679) and Kenya (MK271367) embedded themselves in this clade of *A. caninum* (Figure 4A). For the entries of *N. americanus* ITS, they formed 2 sister clades. The new sequence (PX994166) formed 1 clade with those in Africa (Central African Republic, Gabon, Sudan and Uganda), America (Brazil and Guatemala) and Asia (India). The clade also included the homologs of *A. braziliense*. The other clade included 3 entries from Africa (Gabon) and Asia (Laos and Japan; Figure 4B).

## Discussion

AGMs, an invasive non-human primate, are equal to the human resident population in number on St Kitts. They greatly affect the local economy and very likely pose a high risk to human health by serving as reservoirs of microbial and parasitic pathogens. In this study, we investigate longitudinal dynamics of STHs, i.e. whipworms and hookworms, in a population of wild AGMs living in human residential areas. The prevalence of whipworm and hookworm detected by a combination of direct smear and PCR was 82.2% (95% CI 71.0–93.4%) and 20.0% (95% CI 8.3–31.7%), respectively, with little fluctuation among 4 seasons and between dry and wet seasons. However, it must be pointed out that there is a large disparity between the fall and other season sample numbers, which might skew data statistical analysis. In an earlier cross-sectional survey, the prevalence of whipworm among AGMs was 92.1% (82/89) determined by double centrifugation and PCR (Yao et al., 2018b), which is approximately 10% higher than the current study. One plausible reason for the difference is that the current longitudinal study focuses on 1 area, whereas the previous cross-sectional study was on AGMs at several locations over St Kitts. Alternatively, the difference is likely due to the detection method of direct smear in the current study and centrifugal flotation in the previous one. Direct smear is not a sensitive method for diagnosing most parasitic infections due to the small amount of faeces used (Foreyt, 1989). It was chosen because frozen faeces were used in the current study, which greatly affects parasite eggs’ capacity to float in flotation solutions.

In this study, we showed that hookworm prevalence was 20.0% in wild AGMs by direct smear and PCR, which is consistent with data on hookworm infection in wild non-human primates. Prevalence of hookworm infection in Rhesus macaques (*Macaca mulatta*) was 10.8% in the central mid-hills of Nepal (Tandan et al., 2023) and 24.5% in the urban area of Kathmandu Valley of Nepal (Adhikari et al., 2023); both studies used direct smear and flotation.

Hookworms in AGMs have not been identified to the species level; prior reports relied on morphology of eggs found in faeces (Mutani et al., 2003; Wren et al., 2016; Cruz et al., 2021), which cannot differentiate 1 hookworm species from another. In the current study, we address this gap by targeting ITS regions via PCR followed by DNA sequencing, which identified hookworms in AGMs as *A. duodenale* and *N. americanus*, the same STHs infecting humans. These data are the first to molecularly confirm that hookworms infecting AGMs are the same 2 species infecting humans in very wide geographical locations. Similarly, *N. americanus* has been found in gorillas (*Gorilla gorilla gorilla*) in Gabon by ITS and *cox1* sequences (Hasegawa et al., 2017) and in Cameroon by ITS2 (Ilík et al., 2023). Interestingly, it is not detected in East African chimpanzees (*Pan troglodytes schweinfurthii*) in Uganda and central chimpanzees (*Pan troglodytes troglodytes*) in Cameroon (Hasegawa et al., 2017; Ilík et al., 2023).

The unique geography and tropical climate of St Kitts sustain year-round STH transmission among wild AGMs, which is clearly demonstrated in the current study. Because AGMs frequently interact with humans, they pose a continuous risk to humans for STH infections without seasonal interruption. To achieve the WHO’s goal of eliminating STH morbidity in pre-school and school-age children on St Kitts, AGMs must be incorporated into integrated One-Health strategies.

In the current study, 35.7% of AGM faecal DNAs failed to amplify AGM housekeeping gene *nadh1* by conventional PCR, which is consistent with previous studies. Total DNA extracted from faeces may contain PCR inhibitors such as bilirubin, bile salts, heavy metals, haemoglobin degradation products and complex polysaccharides that interfere with PCR amplification even at low concentrations (Morgan et al., 1998; Stauffer et al., 2008). Failure rates of conventional PCRs using total faecal DNAs as templates targeting AGM *nadh1* and NADH dehydrogenase subunit 6 of the domestic cat were 16.4% (*n* = 11/67) and 49.3% (*n* = 34/69), respectively (Yao et al., 2018a,b). Therefore, closely monitoring PCR inhibitors in faecal DNAs is highly recommended and should be routinely performed to rule out false negatives.

Faeces may be stored frozen prior to parasite analyses such as direct smear and flotation due to safety concerns for examiners and other logistical reasons. Here, we showed that prolonged storage at −80 °C, following freezing in household freezers, did not significantly alter the morphology of whipworm and hookworm eggs, including size, colour and shape (Figure 3), which is different from earlier observations. Damage to the *Parascaris equorum* eggs was only observed in 2% (4/189) eggs. In contrast, 93% (420/450) strongylid eggs were damaged in the horse faeces frozen at −80 °C for 3 days (Schurer et al., 2014). The same authors further observed little damage to *Toxocara canis* and *Toxascaris leonina* and 93% (110/120) damage to the *Uncinaria stenocephala* eggs post freezing in canine faeces (Schurer et al., 2014). Their data suggest that deep freezing has a small effect on ascarid eggs, whereas it has detrimental effects on strongylid/hookworm eggs. Different eggs have different morphologies and structures, causing them to react differently to freezing conditions. Hookworms are likely to be comparable to strongylid eggs as they have a similar structure. The discrepancy between our data and the early ones might be due to differences in freezing protocols. Our samples were frozen in a household freezer for 1–2 months followed by −80 °C, whereas theirs were frozen directly in −80 °C. This needs to be investigated in further studies.

Eggs of both STHs failed to float even in Sheather’s sugar solution by centrifugal flotation after being frozen at −80 °C, which is consistent with earlier observations on hookworm eggs. Nevertheless, little information is available on whipworms. Eggs of *U. stenocephala* floated very poorly, whereas those of *T. leonina* performed well in deep frozen (−80 °C) coyote faeces (Liccioli et al., 2012). Storage at −20 °C and/or −80 °C of canine and equine faeces has a substantial effect on egg flotation capacity of naturally infected animals, i.e. yielding much lower mean egg counts for strongylid eggs and *P. equorum* eggs in horses and *U. stenocephala* eggs in dogs (Schurer et al., 2014). Egg counts of strongylid eggs in horse faeces after being frozen at −10 °C to −18 °C for 1–3 days decreased significantly compared to those kept at room temperature by centrifugal flotation (Nielsen et al., 2010). Collectively, nematode eggs greatly lose their capacity of flotation after freezing. Therefore, frozen faeces should not be analysed by flotation. They can be analysed by direct smear and molecular techniques such as PCR. However, egg morphology may be changed, making identification more challenging.

In conclusion, this longitudinal study has confirmed our hypothesis that STHs are transmitted year-round in AGMs on St Kitts. Furthermore, this study provides the first molecular identification of hookworm species infecting AGMs as *A. duodenale* and *N. americanus*, the human hookworms. Consequently, AGMs pose a continuous risk to human health without seasonal interruption and complicate effort to achieve the WHO’s goal of elimination of STH morbidity in pre-school and school-age children by 2030. Integrating AGMs into One-Health strategies is essential for their effective control on this Caribbean island.

## Supporting information

10.1017/S0031182026102212.sm001 Teng et al. supplementary material 1 Teng et al. supplementary material

10.1017/S0031182026102212.sm002 Teng et al. supplementary material 2 Teng et al. supplementary material

## Data Availability

The DNA sequences have been deposited into the GenBank with the accession numbers PX651763 and PX994166. All data are presented in the manuscript and supplementary materials.

## Supplementary material

The supplementary material for this article can be found at https://doi.org/10.1017/S0031182026102212.

## Figure 1 Long description

The map displays the islands of St. Kitts and Nevis in the Caribbean. St. Kitts is the larger island to the north and Nevis is the smaller island to the south. Various locations are labeled, including Basseterre, which is marked with a red pin. Other labeled areas on St. Kitts include St. Pauls, Tabernacle, Cayon and Sandy Point. On Nevis, locations such as Newcastle, Charlestown and Gingerland are labeled. The map is sourced from Google Maps, with standard map controls visible at the bottom right corner.

Navigate back to Figure 1.

## Figure 2 Long description

Image A shows four photographs of African green monkeys. The first photo depicts a monkey in a tree, surrounded by leaves. The second photo shows several monkeys on a grassy area near a building. The third photo captures a monkey sitting among tree branches. The fourth photo displays a monkey standing on a balcony railing. Image B consists of two photographs of faeces on concrete surfaces. The first photo shows a single faecal deposit, while the second photo displays another faecal deposit, both on similar concrete backgrounds.

Navigate back to Figure 2.

## Figure 3 Long description

The image A showing a left-side group with the label “A” at the upper left. This group contains a grid of small rectangular micrographs. Each micrograph contains one oval object with a darker outline, in brown, dark brown, or pale yellow tones, on a light gray background with scattered small specks. Several ovals are tilted at different angles. Some tiles include additional faint circular or granular material near the oval. The image B showing a left-side group with the label “B” below the left “A” group. This group contains a grid of small rectangular micrographs. Each micrograph contains one oval object with a darker outline, in pale gray to light brown tones, on a light gray background with scattered specks. Some tiles include faint circular rings or partial circular forms near the oval. The image A showing a right-side group with the label “A” near the upper left of the right group. This group contains a grid of small rectangular micrographs. Each micrograph contains one oval object with a darker outline, in brown, dark brown, or pale yellow tones, on a light gray background with scattered specks. The ovals are oriented at varying angles. A thin horizontal scale bar is present near the lower middle area of this right-side group. The image B showing a right-side group with the label “B” below the right “A” group. This group contains two small rectangular micrographs. Each contains one oval object with a darker outline in pale gray tones on a light gray background with scattered specks.

Navigate back to Figure 3.

## Figure 4 Long description

A vertically oriented phylogenetic tree is shown in black on a white background, with branching lines extending from left toward many right aligned tip labels. The figure is split into an upper tree and a lower tree, separated by white space. In the upper tree, the right side lists many tip labels beginning with accession style codes followed by species names, including repeated entries for “Ancylostoma caninum,” “Ancylostoma duodenale,” “Ancylostoma ceylanicum,” “Ancylostoma braziliense,” and “Necator americanus,” each followed by a country name. Small numbers appear near internal branch points as bootstrap values, including 97, 71, 77, 90, 99, 86, 84, 83, 88, 98, 100 and 95. At the bottom left of the upper tree, a horizontal scale bar is labeled “0.10.” In the lower tree, another set of branches extends from the left to right aligned tip labels that include multiple “Necator americanus” entries with country names and one “Ancylostoma braziliense USA” label appears among them. Bootstrap values are printed near internal nodes in this lower tree, including 99, 75, 99, 97, 99 and 100. At the bottom left of the lower tree, a small scale indicator is shown with “2.00” printed nearby.

Navigate back to Figure 4.

## Table 1 Long description

The table summarizes conventional PCR assay characteristics for four organisms, listing target gene, primer sequences, expected amplicon size in base pairs, PCR mix type, thermal cycling, and literature source. African green monkey targets nadh1 with a 348 bp product using mix T1 and cycling with 35 cycles and annealing at 55 degrees C. Trichuris trichiura targets 18S rRNA with a 498 bp product using mix T2 and the same cycling conditions as the first assay. Necator americanus and Ancylostoma duodenale both target the ITS1 to 2 region and use the same primer pair, producing 483 bp and 380 bp products respectively, both with mix T2. The two hookworm assays differ mainly in expected product size but share a higher annealing temperature of 64 degrees C compared with 55 degrees C for the first two assays. Primer sequences include ambiguity codes in the hookworm primers, which may affect exact matching across strains. References are split between a 2018 study for the first two assays and a 2008 study for the hookworm assays.

Navigate back to Table 1.

## Table 2 Long description

Results are from 45 African green monkeys tested by two direct smear slides per animal for whipworm and hookworm. Whipworm had 35 total positives, giving 77.8% prevalence; smear 1 and smear 2 each detected 8 positives with 77.1% sensitivity, and 19 animals were positive on both slides. Hookworm had 7 total positives, giving 15.6% prevalence; smear 1 detected 4 positives with 85.7% sensitivity, smear 2 detected 1 positive with 42.9% sensitivity, and 2 animals were positive on both slides. Overall, whipworm prevalence greatly exceeded hookworm prevalence, and for hookworm the first smear detected more positives than the second. Sensitivity values reflect detection by a single smear compared with the total positives identified across both smears, so they depend on this two-slide reference rather than an external gold standard. The row labeled “Both” repeats the same counts and percentages as the hookworm row.

Navigate back to Table 2.
