# Supplementary material for: Dynamics of zoonotic soil-transmitted helminth infections in wild African green monkey (Chlorocebus aethiops sabaeus)
Source: Parasitology. 2026 Jun 2;153(7):922–9. doi: 10.1017/S0031182026102212 (PMC13735562; doi:10.1017/S0031182026102212)
Supplement: Supplementary file 1 [file S0031182026102212sup001.docx]

***Molar ratio of primers to DNA templates in conventional PCR***

Three DNA targets of three Soil Transmitted Helminths (STH) i.e., *Trichuris trichiura*, *Ancylostoma duodenale*, and *Necatar americanus* and their corresponding primers were used for this purpose. The targets included *T. trichiura* 18S rRNA gene, and internal transcribed spacers (ITS) of *A. duodenale*. GC content was calculated using a free online GC Content Calculator (<https://en.vectorbuilder.com/tool/gc-content-calculator.html?result=true>, accessed 07/23/2025). For *T. trichiura*, the purified PCR amplicon 18S rRNA gene originated from the isolate 8610 (KX961639, 498 bp, 57.59% GC) was used (Yao et al., 2018). Its concentration in millimolar is equal to: X/(Y*660). X is DNA concentration in ng/µL; Y is the length of the DNA sequence in base pair (bp) with an average 660 Daltons in molecular weight. ITS DNAs of *A. duodenale* isolate TNU 331 (MK271367, 704 bp, 47.73% GC), and *N. americanus* isolate UsaOita1 (LC036563, 1,290 bp, 45.12% GC) were both synthesized by Integrated DNA Technologies, Inc. (IDT, Coralville, Iowa). They were resuspended in TE buffer at 10 fm/µL, which was 10 nM. All three DNAs were used in PCR in a serial of 1:10 dilution. Primer’s concentrations were fixed at 1 µM whereas ratios of primers to DNAs ranged from 1:10^-5^ – 10^-16^ µM in individual PCR using HotStart Taq Plus 2× Master Mix (QIAGEN) (**Supplementary Table 1**). Three independent repeats were performed. The highest dilution, i.e. the lowest amount of DNA with all three repeats positive, was used as positive controls for testing individual fecal DNAs.

***Consistent PCR amplification at the lowest primer:DNA ratio of 1:10^-10^***

We were interested in ratios of PCR primer to DNA template and using DNA at the lowest concentration as positive controls in conventional PCRs. In this experiment, the concentrations of both forward and reverse primers were fixed at 1 µM, whereas those of DNA templates covered 12 orders between 10^-5^ µM to 10^-16^ µM. GC contents of five DNA templates ranged from 45.12% to 57.59%. Positive PCR reactions were achieved at least once among three independent repeats in the entire range of DNA concentrations (**Supplementary** **Figure 1, Supplementary Table 1**). Consistent positive results in all three independent repeats covered DNA concentrations of six orders from 10^-5^ µM to 10^-10^ µM for all five targeted DNAs (**Supplementary Table 1**). Interestingly, one pair of primers targeting homologous DNA sequences of *A. duodenale* and *N. americanus* yielded expected size amplicons at the lowest concentrations of 10^-15^ µM and 10^-13^ µM, respectively and with consistent amplification at 10^-10^ µM for both. These findings demonstrate that conventional PCR reliably detects target DNA across six orders of magnitude from 10^-5^ µM to 10^-10^ µM. Consequently, positive controls were always set at 10^-10^ µM when DNA concentrations were known.

**References**

**Yao C, Walkush J, Shim D, Cruz K and Ketzis J** (2018). Molecular species identification of *Trichuris trichiura* in African green monkey on St. Kitts, West Indies. *Veterinary Parasitology: Regional Studies and Reports*, ***11***, 22-26. <https://doi.org/https://doi.org/10.1016/j.vprsr.2017.11.004>

**Supplementary Table 2:** Positive PCR reactions using serial primer/DNA template ratios^1^

|  | 1 | 2 | 3 | 4 | 5 | 6 | 7 | 8 | 9 | 10 | 11 | 12 |
| --- | --- | --- | --- | --- | --- | --- | --- | --- | --- | --- | --- | --- |
| Primer/DNA^2^ | 10^-5^ | 10^-6^ | 10^-7^ | 10^-8^ | 10^-9^ | 10^-10^ | 10^-11^ | 10^-12^ | 10^-13^ | 10^-14^ | 10^-15^ | 10^-16^ |
| A-ITS (380bp)^3^ | 3 | 3 | 3 | 3 | 3 | 3 | 2 | 3 | 3 | 1 | 1 | 0 |
| N-ITS (483bp)^3^ | 3 | 3 | 3 | 3 | 3 | 3 | 3 | 1 | 1 | 0 | 0 | 0 |
| T-18S rRNA (498bp)^3^ | 3 | 3 | 3 | 3 | 3 | 3 | 3 | 1 | 0 | 0 | 1 | 2 |

^1^: The numbers are positive reaction out of three independent repeats.

^2^: PCR primers were fixed at 1 µM whereas the concentrations of DNA templates were 10^-5^ to 10^-16^ µM in individual PCR.

^3:^ A: *Ancylostoma duodenale*; N: *Necator americanus*; T: *Trichuris trichiura*.

**Supplementary Figure 1**: PCR amplification using different primer/DNA template ratios. One of three independent repeats is shown. L: 100 bp DNA ladder with sizes marked on the left. Lanes 1-12 represents 10^-5^ to 10^-16^ µM DNA template in a 1:10 serial dilution, respectively, under the same primer concentrations at 1 µM. **a**: *Ancylostoma* internal transcribed spacer (ITS) – 380 bp; **b**: *Necator* ITS – 483 bp; **c**: *Trichuris* 18S rRNA – 498 bp.
