# Supplementary material for: Dynamics of zoonotic soil-transmitted helminth infections in wild African green monkey (Chlorocebus aethiops sabaeus)
Source: Parasitology. 2026 Jun 2;153(7):922–9. doi: 10.1017/S0031182026102212 (PMC13735562; doi:10.1017/S0031182026102212)
Supplement: Supplementary file 2 [file S0031182026102212sup002.docx]

**Supplementary Table 1**. Monthly average temperature on St. Kitts

| Average | **Jan** | **Feb** | **Mar** | **Apr** | **May** | **Jun** | **Jul** | **Aug** | **Sep** | **Oct** | **Nov** | **Dec** |
| --- | --- | --- | --- | --- | --- | --- | --- | --- | --- | --- | --- | --- |
| High | 83°F | 83°F | 83°F | 84°F | 85°F | 86°F | 87°F | 87°F | 87°F | 87°F | 85°F | 83°F |
| Temp. | 78°F | 78°F | 78°F | 80°F | 81°F | 82°F | 83°F | 83°F | 83°F | 82°F | 81°F | 79°F |
| Low | 75°F | 74°F | 75°F | 76°F | 78°F | 79°F | 80°F | 80°F | 79°F | 79°F | 77°F | 76°F |

From <https://weatherspark.com/y/150244/Average-Weather-in-Saint-Kitts-and-Nevis-St.-Kitts-&-Nevis-Year-Round> (Accessed September 2, 2025)
